# Deubiquitinating Enzyme Inhibitors Block Chikungunya Virus Replication

**DOI:** 10.3390/v15020481

**Published:** 2023-02-09

**Authors:** Lady S. López, Eliana P. Calvo, Jaime E. Castellanos

**Affiliations:** Grupo de Virología, Universidad El Bosque, Bogotá 110121, Colombia

**Keywords:** chikungunya virus, infection, replication, ubiquitination, deubiquitinating enzymes, deubiquitinase inhibitor

## Abstract

Ubiquitination and deubiquitination processes are widely involved in modulating the function, activity, localization, and stability of multiple cellular proteins regulating almost every aspect of cellular function. Several virus families have been shown to exploit the cellular ubiquitin-conjugating system to achieve a productive infection: enter the cell, promote genome replication, or assemble and release viral progeny. In this study, we analyzed the role of deubiquitinating enzymes (DUBs) during chikungunya virus (CHIKV) infection. HEK293T, Vero-E6, and Huh-7 cells were treated with two DUB inhibitors (PR619 or WP1130). Then, infected cells were evaluated by flow cytometry, and viral progeny was quantified using the plaque assay method. The changes in viral proteins and viral RNA were analyzed using Western blotting and RT-qPCR, respectively. Results indicate that treatment with DUB inhibitors impairs CHIKV replication due to significant protein and viral RNA synthesis deregulation. Therefore, DUB activity may be a pharmacological target for blocking CHIKV infection.

## 1. Introduction

Ubiquitination is a post-translational modification that involves the covalent binding of one or more ubiquitin (Ub) molecules to a target protein, which leads to changes in its function, location, activity, or stability [1]. Ub conjugation is a sequential, three-step process that begins with the activation of the Ub molecule by an activating enzyme (E1). Next, a conjugating enzyme (E2) transports Ub to the substrate protein. The final step involves the formation of an isopeptide bond between the terminal α-carboxyl group of ubiquitin and the ε-amino group of a lysine (K) present in the target protein catalyzed by E3 ligases [2]. The presence of seven lysine residues (K6, K11, K27, K29, K33, K48, and K63) in ubiquitin allows the formation of different Ub chains (poly-Ub), and the type of link between the Ub molecules defines the route or destination of the ubiquitinated protein. For example, the Poly-Ub K-48 label is a signal for protein degradation by the proteasome. The ubiquitin–proteasome system (UPS) is the primary pathway regulating protein turnover and contributes to cellular homeostasis [3].

Ubiquitination is a dynamic process that is reversible due to deubiquitinating enzymes (DUBs), which hydrolyze poly-Ub chains, remove Ub from labeled proteins, and process Ub precursor proteins. The human genome encodes nearly 100 DUBs, which are classified into seven families according to their structure and mechanism of catalysis. Six are cysteine proteases, and the seventh family includes metalloenzymes. Ubiquitin-specific proteases (USPs) are the largest and most diverse family, with 56 members, followed by ovarian tumor proteases (OTUs), with 17 members, and the JAMM family (metalloenzymes containing the Jad1/Pad/MPN domain), with 12 members. The MINDY family, Machado Joseph disease protease family (MDJ), ubiquitin terminal carboxyl hydrolases (UCHs), and the newly discovered ZUFSP/Mug105 family are less common [4].

Fundamental cellular processes, such as cell cycle progression, endocytosis, intracellular trafficking, DNA repair, transcription, signaling, and antigen presentation, are regulated through ubiquitination, deubiquitination, and the UPS system [5]. Various viruses use this system to evade the immune response or to achieve productive infection [6].

Multiple studies have shown that some alphaviruses, flaviviruses, retroviruses, coronaviruses, and herpes viruses require ubiquitination and proteasome activity in the early stages of infection (entry and uncoating) or for other crucial processes, such as viral genome synthesis, viral protein translation, assembly, maturation, and the release of viral progeny [6,7,8,9].

Analysis of the changes in the cellular proteome caused by infection with chikungunya virus (CHIKV) suggests that the UPS system is essential during infection in both cell cultures and murine models [10,11,12]. These studies showed significant changes in proteins involved in ubiquitination, deubiquitination, and the proteasome. For example, the conjugating enzymes UBE2N and UbcH10, the E3 ligase RBX1, the DUB UBXN6, and the α-6 subunit of the proteasome (PSMA6) are downregulated during infection. In contrast, the E3 ligase RNF213, the adaptor protein RAD23A, and the DUB USP13 are upregulated [10,11,12]. On the other hand, Karpe et al. reported the requirement of a functional proteasome for CHIKV replication. In GripTite-293 MSR cells, proteasome inhibition decreased viral progeny, and the accumulation of polyubiquitinated proteins induced the unfolded protein response (UPR) [13]. BHK-21 and HeLa cells treated with the proteasome inhibitor bortezomib during the early phase of infection also showed decreased viral production due to a defect in viral protein synthesis [14].

CHIKV is an alphavirus of the family *Togaviridae* and the causative agent of a febrile illness accompanied by rash, headache, myalgia, and severe joint pain [15]. Arthritis is the main complication associated with CHIKV infection; disabling and recurrent pain can occur months or years after the acute phase, which reduces the quality of life and has a significant economic impact because it affects work capacity [16,17]. During the first outbreak in the American continent in 2013–2016, there was not only a rapid spread of the virus and a high number of documented cases [18] but also a large number of cases of severe disease, with atypical manifestations and a considerable increase in mortality in Brazil, the Dominican Republic, and Guadeloupe, three of the five most affected countries on the continent [19].

No vaccine or specific drugs have been approved for the treatment of CHIKV. In a search for new antiviral strategies against CHIKV, the requirement for DUB activity during CHIKV infection, which is yet unknown, was analyzed by evaluating the effect of selective inhibitors of DUBs on viral replication.

## 2. Materials and Methods

### 2.1. Cells and Virus

The adherent cell lines HEK293 (ATCC^®^ CRL-11268G-1™), Vero clone E6 (ATCC^®^ CRL-1586™), Huh-7 (JCRB0403) and BHK-21 (ATCC^®^ CCL-10™) (ATCC, Manassas, VA, USA) were grown in Dulbecco’s modified Eagle’s medium (DMEM; Sigma–Aldrich, St Louis, MI, USA) supplemented with 10% fetal bovine serum (FBS; GIBCO, Carlsbad, CA, USA) at 37 °C and 5% CO_2_. The CHIKV isolate used in this study, COL7624, was collected in Colombia in 2015 and passaged four times in Vero cells before titration (GenBank ID: MH329298.1). The characterization of this viral isolate, including its ability to infect and replicate in HEK293 and Huh-7 cells, has already been reported by Archila et al., 2022 [20].

### 2.2. Reagents and Antibodies

The DUB inhibitors PR-619 (Cat#662141) and WP1130 (Cat#681685) were purchased from Calbiochem, San Diego, CA, USA and were used at the concentrations indicated in the different experiments. Mouse polyclonal antibody directed against the E1 protein was provided by Anne Claire Brehin (Institut Pasteur, Paris, France), and rat polyclonal serum against nsP1 was obtained after injection of a recombinant protein produced in our laboratory. For Western blotting and flow cytometry, the following antibodies were used: mouse monoclonal anti-CHIKV virus antibody (ab155841, Abcam, Cambridge, MA, USA), anti-actin mAb (Cat. # 8457, Cell Signaling Technology CST, Danvers, MA, USA), and the DUB Antibody Sampler Kit including anti-rabbit mAbs against HAUSP, UCHL1, A20/TNFAIP3, USP10, STAMBP, CYLD, and pCYLD proteins (Cat. # 8353, CST). Horseradish peroxidase (HRP)-conjugated anti-mouse (12-349), anti-rat (12-349), and anti-rabbit (12-348) secondary antibodies were purchased from Sigma–Aldrich.

### 2.3. Inhibitor Treatment, Cell Viability Evaluation, and CHIKV Infection

Cells were seeded in 96-well plates, and after overnight incubation, the medium was removed and replaced with 100 µL of fresh medium containing either 0.1% DMSO or a DUB inhibitor at the indicated concentration and incubated for 24 h. Then, resazurin solution (44 µM) was added, and the plate was incubated at 37 °C for 2 h. Finally, the fluorescent signal was read using a Tecan Infinite 200 fluorometric reader (Tecan Trading AG, Männedorf, Switzerland) at excitation and emission wavelengths of 535 and 595 nm, respectively.

HEK293, Vero-E6, and Huh-7 cells were seeded at densities of 100.000–200.000 cells per well and incubated overnight at 37 °C. The next day, monolayers were incubated with PR-619, WP1130, or 0.1% DMSO for 1 h. Treated cells were then infected with CHIKV at MOI 1 or 0.5 for 1 h. Finally, infected cell monolayers were incubated in DMEM containing 2% FBS and inhibitor for 24 h at 37 °C. Supernatants were collected for titration, and cells were fixed for antigen detection using flow cytometry.

For the post-infection assay, cell monolayers were infected with CHIKV for 1 h at 37 °C. Then, the inoculum was removed, and the cells were incubated in DMEM with 2% FBS, followed by the addition of DUB inhibitor. Finally, at 24 h post-infection (hpi), supernatants were collected for titration, cells were collected for antigen detection using flow cytometry, RNA was prepared for viral RNA quantification using RT-qPCR, and viral proteins were analyzed using Western blotting (Figures S1–S3).

### 2.4. Infected Cell Detection Using Flow Cytometry

Cells were permeabilized using Cytofix/Cytoperm (BD Biosciences, Franklin Lakes, NJ, USA), incubated with anti-E1 antibody (Abcam) diluted 1:50 in Perm/Wash buffer (BD Biosciences), and then stained with secondary FITC-conjugated anti-mouse Ab (Sigma). Flow cytometry was performed using a BD Accuri™ C6 cytometer (BD Biosciences). Data were analyzed using FlowJo™ Software (BD Biosciences).

### 2.5. Viral Protein Detection Using Western Blotting

Cells were lysed in RIPA buffer containing a protease inhibitor cocktail for 30 min at 4 °C. Protein samples were separated by SDS-PAGE and then transferred onto polyvinylidene fluoride (PVDF) membranes (GE Healthcare, Chicago, IL, USA). Membranes were blocked with 5% non-fat milk in TBST and incubated with the appropriate primary antibody for 1 h. For DUB detection, the membranes were blocked with 3% bovine serum albumin in TBST. After extensive washing, the membrane was incubated with each antibody and then with anti-mouse, anti-rat, or anti-rabbit secondary antibodies coupled to horseradish peroxidase (HRP). The blots were developed using West Pico Blotting Detection Reagent (Thermo Fisher Scientific, Waltham, MA, USA), and images were captured using a Gel Doc system (BioRad, Hercules, CA, USA).

### 2.6. Viral RNA Quantification Using RT-qPCR

Total RNA was extracted from infected cells using the PureDireX Total RNA Isolation Kit (Bio-Helix, New Taipei City, Taiwan) according to the manufacturer’s instructions. The eluted RNAs were quantified, normalized to 20 ng/mL, and stored at −80 °C until use. Samples were assayed with a one-step RT-qPCR protocol using the Luna^®^ Probe system (New England Biolabs, Ipswich, MA, USA) and the following primers targeting the *nsp4*, *E1*, and actin as a reference gene for normalization: nsp4F, TCACTCCCTGTTGGACTTGATAGA; nsp4R, TTGACGAACAGAGTTAGGAACATACC; nsp4P Texas Red/AGGTACGCGCTTCAAGTTCGGCG/BHQ2; E1F, AAGCTYCGCGTCCTTTACCAAG; E1R, CCAAATTGTCCYGGTCTTCCT, E1P FAM/CCAATGTCYTCMGCCTGGACACCTTT/BHQ-1; actin F, GGATGCAGAAGGAGATCACTG; actin R, CGATCCACACGGAGTACTTG; and actin HEX/CCCTGGCACCCAGCACAATG/BHQ1. Each reaction (20 μL) contained 10 μL of 2× master mix, 200 nM primers, 200 nM probes, and 60 ng of RNA. Reactions were performed in a CFX96 real-time PCR cycler (BioRad) under the following cycling conditions: 15 min of reverse transcription at 55 °C, followed by a denaturation step at 95 °C for 3 min, and 40 cycles of 95 °C for 15 s and 60 °C for 1 min. The fold changes in the inhibitor-treated samples, when compared to the levels in the untreated samples, were calculated using Pfaffl’s (2001) method [21].

### 2.7. Viral Titration Using Plaque Assay

BHK-21 cells were seeded at a density of 80.000 cells/well in 24 well plates. The next day, confluent monolayers were infected with 10-fold dilutions of the samples prepared in 2% media. After one hour of incubation, media containing DMEM (Sigma-Aldrich, St. Louis, MS, USA), 3% CMC, 7.5% sodium bicarbonate (NaHCO_3_) (Sigma-Aldrich), supplemented with FBS and antibiotic was added; cells were incubated for 72 h. Then, cells were fixed and stained with 1% crystal violet (Sigma-Aldrich). The number of plaques was counted and is expressed as plaque-forming units per milliliter (PFU/mL).

### 2.8. Statistical Analysis

Individual experiments were performed in triplicate, and the results are shown as the arithmetic mean and standard deviation. One-way analysis of variance (ANOVA) followed by Dunnett’s post-hoc test was performed to determine the significance of differences between the experimental groups and the controls using GraphPad Prism 7. A *p*-value less than 0.05 was considered significant.

## 3. Results

### 3.1. Chikungunya Virus Requires the Activity of DUBs to Achieve a Productive Infection

**Figure 1:**
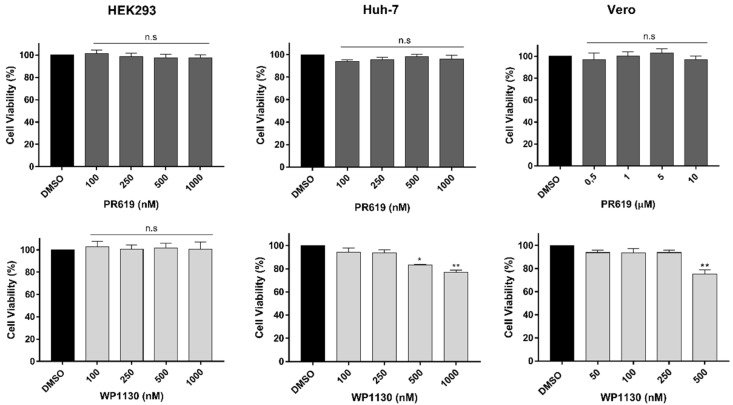
Cytotoxic effect of deubiquitinating enzyme inhibitors. HEK293, Huh-7, and Vero cells were exposed to PR-619 or WP1130 at the indicated concentrations, DMSO 0.1% was used as a control. After 24 h of treatment, cell viability was analyzed using the resazurin assay method. Data are represented as the mean + SD, and statistically significant differences are indicated as * *p* < 0.05, ** *p* < 0.01. *n.s* no significant differences.

This study investigated the requirement of deubiquitinating activity during CHIKV infection using specific inhibitors that selectively modulate DUB activities with a relatively low range of toxicity and off-target activities. PR-619 is selective for USP and UCH families over other cysteine proteases, including phospholipase A, calpain 1, cathepsin B, cathepsin D, matrix metallopeptidase 13, and trypsin [22]. While, WP1130 mainly inhibits USP9X, USP5, USP14, and UCHL37 [23]. To evaluate the natural cytotoxicity of the inhibitors, cells were exposed to different concentrations of the inhibitors for 24 h, and then, cell viability was analyzed. In HEK293 cells, the addition of the inhibitors in a range of 50–1000 nM had no deleterious effect; cell viability remained greater than 90% at the tested concentrations (Figure 1). However, a different result was obtained for Vero and Huh-7 cells; while viability was >90% in cells exposed to 50 and 250 nM WP1130, exposure to 500 nM or higher concentrations resulted in a 25% decrease in the number of viable cells. Light microscopy revealed a loss of characteristic morphology, rounded cells, and monolayer detachment. In contrast, exposure to PR-619, even at concentrations of 5 and 10 μM, did not significantly alter cell viability (Figure 1). Based on these results, in subsequent experiments, both PR-619 and WP1130 were used at 1μM for HEK293 cells, 5μM PR-619 for Vero, 1μM for Huh-7 and 250 nM WP1130 for Vero and Huh-7 cells.

**Figure 2:**
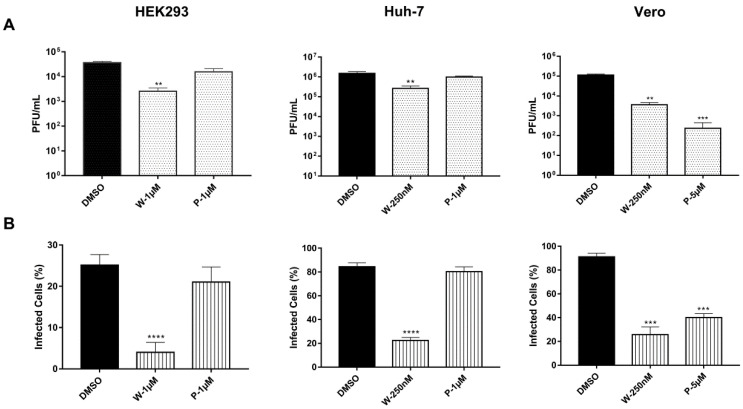
Inhibition of deubiquitinating enzymes impairs chikungunya virus replication. Cells were pre-treated with the indicated concentrations of WP1130 (W), PR-619 (P), or DMSO for 1 h. After that, cells were infected with CHIKV for 1 h, the inoculum was then removed and replaced with fresh media supplemented with inhibitors. After 24 h of infection, supernatants and cells were collected. (**A**) Released viral progeny was measured using the plaque assay method. (**B**) Infected cells were analyzed using flow cytometry. Data are represented as the mean + SD, and statistically significant differences are indicated as ** *p* < 0.01, *** *p* < 0.001, **** *p* < 0.0001.

To assess the effect of DUB inhibition on productive virus infection, cells were treated with the inhibitor, infected with the virus, and kept in the presence of the inhibitor for a further 24 h. Then, the infectious viral progeny released into the supernatant was quantified using plaque assays in BHK-21 cells. Treatment with WP1130 caused a significant decrease in viral progeny in all the tested cell types. Similar behavior in Huh-7 and HEK293T cells was evidenced since the viral titer fell by 1 log unit (~10 times lower) and 1.1 log units (~12.5 times lower), respectively. In contrast, Vero cells displayed the most significant reduction of 1.4 log units (~25 times lower) in viral yield. Treatment with PR-619 had different effects. In HEK293T and Huh-7 cells, no significant changes in viral titer were observed, whereas in Vero cells, viral titer was significantly reduced by 2.5 logarithmic units (>300-fold reduction; Figure 2A). In addition, evaluation of intracellular viral antigen by flow cytometry showed that WP1130 treatment drastically affected infection in all the cell types, and the number of infected cells was reduced by ~70%. In contrast, treatment with PR-619 only affected Vero cells, where the number of infected cells decreased by ~60% (Figure 2B). Taken together, these data indicate that DUB inhibition interferes with CHIKV replication.

**Figure 3:**
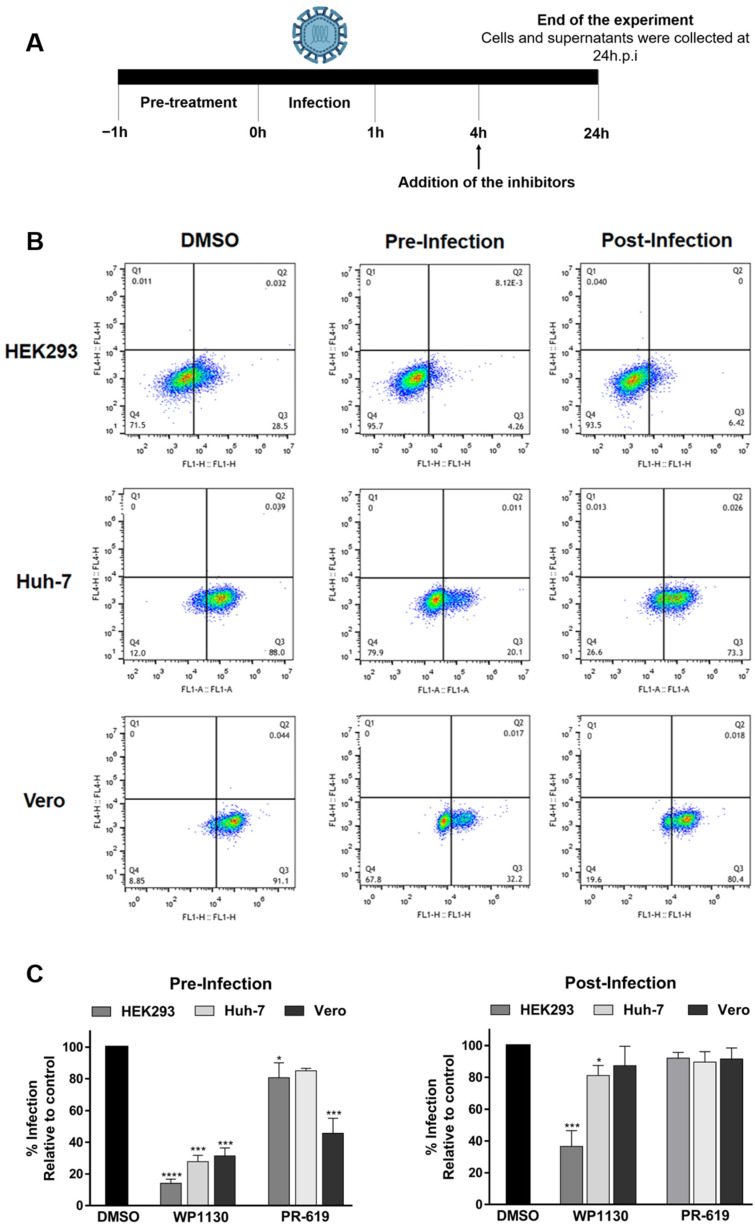
Deubiquitinating activity is required at an early stage of infection. (**A**) Schematic representation of the experiment. (**B**) Cells were treated with WP1130 at the indicated concentrations 1 h before infection or 4 h post-infection. Cells were collected 24 h later and analyzed using flow cytometry. (**C**) Infection rates normalized to control (DMSO). Data are represented as the mean + SD, and statistically significant differences are indicated as * *p* < 0.05, *** *p* < 0.001, **** *p* < 0.0001.

To determine whether deubiquitinating activity is required during the early phase of the replication cycle, inhibitor treatment was initiated either before infection (pre-infection) or 4 h after virus inoculation (post-infection), and viral antigen was detected at 24 hpi (Figure 3A). Cell lines showed different infection profiles; HEK293 cells were infected by 25%, whereas Vero and Huh-7 cells displayed higher infection rates (~80%) (Figure 3B). Treatment with WP1130 before infection significantly decreased infection rates by more than ~70% in all the cell lines (Figure 3C). However, adding the inhibitor after infection only reduced the percentage of infected HEK293T cells. Treatment with PR619 pre- or post-infection did not significantly affect the number of human-infected cells; in contrast, Vero cells showed a considerable reduction in the percentage of CHIKV-positive cells when the inhibitor was added before virus inoculation (Figure 3C). Altogether, these results suggested that deubiquitinating activity is fundamental to CHIKV replication and that DUBs regulate critical events during the early phase of infection.

### 3.2. Inhibition of DUBs Affects Key Events in Viral Replication

**Figure 4:**
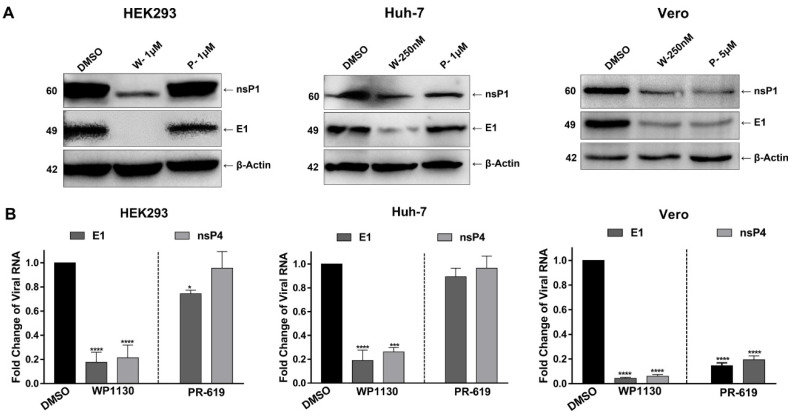
Inhibition of deubiquitinating enzymes impairs protein and viral RNA synthesis. Cells were pre-treated with the inhibitors WP1130 (W) or PR-619 (P) at the indicated concentrations, then infected with CHIKV for 1 h; after this time, the inoculum was removed and replaced with fresh media supplemented with inhibitors. After 24 h of infection, cells were collected. (**A**) Viral protein levels were evaluated using Western blot with antibodies against E1 and nsP1. β-actin was used as a loading control. (**B**) Levels of intracellular viral RNA were measured using RT-qPCR. Data are represented as the mean + SD, and statistically significant differences are indicated as * *p* < 0.05, *** *p* < 0.001, **** *p* < 0.0001.

To assess whether the decrease in the infective viral progeny was due to a defect in viral protein synthesis or genome replication events in the early stage of the viral replication cycle; we evaluated the levels of both structural proteins, which are indispensable for virion formation, and nonstructural proteins, which are essential for genome replication, in cells treated with the inhibitors. In infected cells treated with DMSO, antibodies recognized a 49 kDa band corresponding to E1 and a 60 kDa band corresponding to nsP1 (Figure 4A). Exposure to the inhibitor led to a drastic reduction in the levels of both proteins; in HEK293T cells, the signal of the E1 protein disappeared completely after treatment with WP1130. In Vero and Huh-7 cells, a similar behavior was evidenced; levels of E1 protein were significantly reduced during WP1130 treatment, while nsP1 protein was reduced to a lesser extent. Notably, in Vero cells, PR-619 treatment affected the levels of both viral proteins. These results are consistent with the detection of low levels of E1 in cells treated with the DUB inhibitors using flow cytometry and could explain the decrease in the viral progeny titer (Figure 2).

E1 and E2 are glycoproteins located in the virus envelope that allow interaction with the host cell at different phases of viral entry, such as target cell adhesion, internalization, intracellular trafficking, and fusion of membrane proteins [24]. In contrast, nsP1 has methyltransferase and guanyl-transferase activities, and it is responsible for the formation of the 5′-cap on genomic RNA (gRNA), a structure required to protect RNA from degradation by cellular exonucleases and structural elements located in the 5′-UTR and for the translation of viral proteins [25]. Thus, alteration in the production of E1/E2 has direct consequences on the assembly of viral particles and indirect implications on virion entry. In contrast, alteration of nsP1 levels could have repercussions on the stability of the viral genome.

To analyze the effect of DUB inhibition on the synthesis of viral RNA (vRNA), vRNA levels were quantified using RT-qPCR. Beta-actin was used as a reference gene, and cells treated with DMSO as a sample control. There was a significant reduction in vRNA levels in inhibitor-treated cells. A substantial decrease was observed in all the cell lines treated with WP1130. Exposure to PR619 resulted in a 5-fold reduction in vRNA levels in Vero cells (Figure 4B). These results confirmed that inhibition of DUBs affects the early phase of infection, altering both the translation of viral proteins and the synthesis of the viral genome, which directly impacts the generation of viral progeny.

### 3.3. Deubiquitinating Enzymes Are Not Regulated during CHIKV Infection

**Figure 5:**
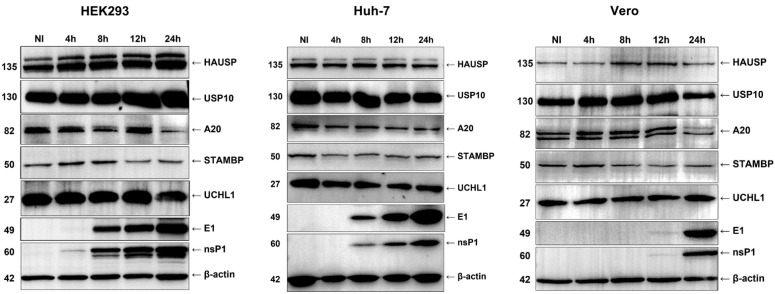
Expression of deubiquitinating enzymes throughout CHIKV infection. Cells were infected with CHIKV for 1 h; the inoculum was removed and replaced with fresh media; then, cells were collected at the indicated times. Expression of different DUB proteins was assessed using Western blot with antibodies against HAUSP, USP10, A20, STAMBP, and UCHL1, whereas viral protein levels were evaluated using antibodies against E1 and nsP1. β-actin was used as a loading control.

Several studies have shown the overexpression or downregulation of some DUBs during viral infection [26,27,28,29]. To determine whether DUB protein expression was modified during CHIKV infection, a kinetic analysis was performed, and a panel of six proteins was evaluated (Figure 5). Viral proteins (E1 and nsP1) were detectable as early as 4 h post-infection in HEK293 cells, 8 hpi in Huh-7 cells, and 12 hpi for Vero cells. Regarding DUB expression, no apparent differences were found throughout infection in the levels of HAUSP, also called USP7, UCHL1, A20, USP10, STAMBP, and CYLD. Phosphorylated or activated CYLD was not detected.

## 4. Discussion

In the last decade, CHIKV has re-emerged as a major public health threat due to frequent infections, which result in high morbidity and economic costs [17]. Currently, no specific drugs exist to treat CHIKV infections, and potential vaccine candidates are still under investigation. Therefore, there is a need to explore new therapeutic targets for the treatment and rapid control of CHIKV infection.

DUBs cleave multiple types of poly-UB chains, thereby modulating critical processes occurring inside the cell that are regulated by protein ubiquitination. In the present study, we evaluated the role of deubiquitination during CHIKV infection using two DUB inhibitors (PR-619 and WP1130). PR-619 is a reversible inhibitor that induces endoplasmic reticulum (ER) stress and related apoptosis and is a broad-spectrum DUB inhibitor that targets nine members of the USP family (2/4/5/7/8/15/20/28/47) and three of the four members of the UCHL family (1/3/5) in human cells. In contrast, WP1130 is a specific inhibitor of USP5, 9X, 14, 37, and UCHL5. It is currently in preclinical studies as a drug against melanoma and glioblastoma [30].

The experiments were performed in three cell lines with different susceptibilities to infection. HEK293T cells reached moderate infection percentages of 25%, whereas Vero and Huh-7 cells reached infection percentages of 80–90% at 24 hpi. Decreasing deubiquitinating activity by treating cells with WP1130 led to significant reductions in infected cells and infective progeny in all the cell lines tested. In contrast, exposure to PR-619 did not affect viral replication in HEK293T and Huh-7 cells. These results indicate that DUB activity is fundamental to the CHIKV replicative cycle. However, these data suggest differences between the analyzed cell types in either the active DUBs and those involved in viral replication. The blocking activity of WP1130 is restricted to five DUBs; however, two of these enzymes, USP5 and UCHL5, are also targets of PR-619, which did not affect productive infection. Another target of WP1130 is USP14, which can be blocked by a specific inhibitor called IU1, which has previously been shown to have no effect on CHIKV replication in HEK293T cells, even at concentrations of 75–100 µM [31]. Therefore, we hypothesized that either USP9X or USP37 might play a determining role in CHIKV replication in HEK293T cells. However, further research is necessary to confirm these findings in other cell lines.

Some authors have previously reported a requirement for DUBs during viral infection. Nag and Finley showed that IU1 interferes with the replication of some flaviviruses, such as West Nile (WNV), yellow fever virus (YFV), and dengue (DENV), with the most potent effect on DENV [31]. Perry et al. (2012) found that infection with human and murine norovirus, Sindbis virus, La Crosse virus, and encephalomyocarditis virus was drastically decreased in the presence of WP1130 [32]. They also showed that the antiviral effect was related to the unfolded protein response (UPR) activation, which is turned on by inhibiting DUBs. More recently, Setz et al. (2017) reported a 90% reduction in the replicative capacity of the human immunodeficiency virus (HIV-1) in lymphoid cells after treatment with PR-619 [33]. Interestingly, Große et al. (2022) found that DUB inhibitors interfere with SARS-CoV2 Papain-like protease enzymatic activity in a dose-dependent manner, thus impairing viral replication [34].

Our findings demonstrate that DUB activity is essential during the early phase of CHIKV infection. However, DUB activity could also modulate late events in the CHIKV replication cycle (Figure 3). Processes such as the translation of viral proteins and synthesis of the viral genome were affected by DUB inhibitors (Figure 4). Several studies have reported alterations in viral proteins following exposure to PR-619. In HIV-1, the processing of Gag polyprotein, which contains the structural proteins required to form the viral particle, including matrix, capsid, and nucleocapsid proteins, was blocked [33]. The stability of Tat protein, which functions to enhance HIV-1 replication, was also reduced [35]. In contrast, WP1130 treatment during norovirus infection in vitro reduced genome replication, whereas earlier stages of infection, namely receptor attachment, and viral entry, were not impaired [32].

Finally, the expression of six DUBs was evaluated during CHIKV infection. USP7, USP10 and CYLD, members of the USP family; UCHL1, which belongs to the UCHL family; STAMBP, which is a JAMM family metalloprotease; and otubain A20. These enzymes are involved in signaling, DNA repair, transcription, and endocytosis, and some of them, such as A20 and CYLD, contribute to establishing the antiviral status of cells [4]. During CHIKV infection, no apparent changes in the levels of these proteins were observed in HEK293T, Huh-7, or Vero cells (Figure 5). These results differ from previous reports showing that measles virus and human papillomavirus (HPV) infection led to the overexpression of A20 and UCHL1, respectively [29,30]. Increased levels of A20 in the monocytic cell lines U937 and THP-1 modified the ubiquitination state of TRAF6, blocking NF-κB activation [30]. In HPV-infected keratinocytes, high levels of UCHL1 altered TRAF3 polyubiquitination, downregulating stages of the immune response, such as the production of interferons and pro-inflammatory cytokines [27]. For the influenza A virus, induced expression of A20 prevented the establishment of an antiviral state in the cell, thus facilitating its replication [26]. Nair et al. reported a higher expression of USP18 in Huh-7 cells infected with CHIKV, being USP18 a DUB involved in the negative regulation of the NF-κB signaling pathway [29].

## 5. Conclusions

This work demonstrates for the first time that cellular DUBs are required for optimal CHIKV infection in mammalian cells. DUB inhibitors impair the synthesis of viral RNA and viral proteins. However, the mechanisms by which DUBs are involved in these processes remain to be defined.

## Data Availability

The data presented in this study are openly available in a public repository.

## Supplementary Materials

The following supporting information can be downloaded at: https://www.mdpi.com/article/10.3390/v15020481/s1.
