# Supplementary material for: Deubiquitinating Enzyme Inhibitors Block Chikungunya Virus Replication
Source: Viruses. 2023 Feb 9;15(2):481. doi: 10.3390/v15020481 (PMC9966916; doi:10.3390/v15020481)
Supplement: Supplementary file 1 [file viruses-15-00481-s001.zip › viruses-2133364-supplementary.pdf]

Fig S1 Cell Viability

	HEK293				
	PR-619 (nM)				
	DMSO	100	250	500	1000
Cell Viability (%)	100	104,69	100,93	99,43	100,53
	100	100,11	99,60	99,22	95,50
	100	98,88	95,56	93,97	96,38
	100	93,10	91,53	99,87	97,51
	100	101,49	98,12	94,22	97,16
	100	98,81	90,42	97,94	97,48

	WP1130 (nM)				
	DMSO	100	250	500	1000
Cell Viability (%)	100	104,60	104,61	103,31	104,23
	100	106,09	100,21	104,48	104,26
	100	97,00	97,20	96,64	93,65
	100	100,81	97,27	98,46	99,11
	100	99,43	99,50	99,19	96,68
	100	98,46	96,69	95,95	95,62

	Huh-7				
	PR-619 (nM)				
	DMSO	100	250	500	1000
Cell Viability (%)	100	92,68	96,80	98,44	98,99
	100	94,26	95,34	99,99	94,47
	100	92,06	91,84	99,49	98,80
	100	94,47	98,49	94,78	94,38
	100	96,08	95,81	96,28	98,79
	100	93,83	94,37	99,36	90,07

	WP1130 (nM)				
	DMSO	100	250	500	1000
Cell Viability (%)	100	97,86	97,37	82,58	76,00
	100	91,24	95,82	82,32	74,04
	100	97,05	91,02	83,22	77,94
	100	95,82	92,58	82,96	77,61
	100	89,33	93,83	84,13	75,61
	100	95,03	92,01	83,86	79,59

	Vero				
	PR-619 (μM)				
	DMSO	0,5	1	5	10
Cell Viability (%)	100	92,70	96,28	93,30	95,82
	100	101,06	95,48	97,84	90,94
	100	95,69	96,15	96,97	93,33
	100	104,04	98,93	91,10	91,93
	100	92,89	91,36	91,56	93,45
	100	94,00	90,33	96,52	91,02

	WP1130 (nM)				
	DMSO	50	100	250	500
Cell Viability (%)	100	92,317	90,968	95,329	76,123
	100	95,325	96,329	92,357	75,967
	100	98,666	97,857	93,895	71,082
	100	101,858	97,935	93,712	76,279
	100	95,639	93,237	96,499	73,211
	100	95,066	97,589	92,289	76,056

Fig S2 Plaque Assay

HEK293

	DMSO	WP1130 1µM	PR619 1µM
PFU/mL	3,5,E+04	3,0,E+03	2,5,E+04
	4,5,E+04	1,0,E+03	1,8,E+04
	4,0,E+04	2,0,E+03	1,5,E+04
	3,5,E+04	2,5,E+03	1,0,E+04
	3,0,E+04	3,5,E+03	1,0,E+04
	3,3,E+04	3,3,E+03	1,5,E+04

Vero

	DMSO	WP1130 250nM	PR619 5µM
PFU/mL	1,3,E+05	5,0,E+03	5,0,E+01
	9,0,E+04	3,5,E+03	1,5,E+02
	1,1,E+05	2,5,E+03	1,0,E+02
	1,1,E+05	4,5,E+03	5,0,E+02
	1,2,E+05	2,5,E+03	5,0,E+02
	1,3,E+05	3,5,E+03	1,0,E+02

Huh-7

	DMSO	WP1130 250nM	PR619 1µM
PFU/mL	1,2,E+06	1,5,E+05	1,1,E+06
	1,2,E+06	2,5,E+05	1,1,E+06
	1,2,E+06	2,0,E+05	1,1,E+06
	7,5,E+05	2,5,E+05	1,0,E+06
	9,0,E+05	4,0,E+05	8,0,E+05
	8,3,E+05	3,3,E+05	9,0,E+05

Fig S2 Flow Cytometry

HEK293

	DMSO	WP1130 1µM	PR619 1µM
Infected Cells (%)	20,6	3,6	24,6
	27,3	1,4	20,0
	24,5	4,3	14,9
	24,6	6,4	24,6
	27,3	7,0	20,0
	26,5	1,4	22,1

Huh-7

	DMSO	WP1130 250nM	PR619 1µM
Infected Cells (%)	88,0	20,1	76,8
	87,9	24,1	74,1
	80,7	24,0	83,6
	84,4	21,4	82,6
	74,7	19,4	84,0
	73,6	25,6	80,8

Vero

	DMSO	WP1130 250nM	PR619 5µM
Infected Cells (%)	91,5	18,1	38,7
	90,1	22,3	39,1
	91,1	32,2	35,5
	88,3	33,9	40,2
	96,8	20,3	41,4
	89,0	27,6	45,5

Fig S3 Flow Cytometry-Phase

HEK293

Infection Rates

DMSO	WP1130 (Pre-Treatment)	WP1130 (Post-Treatment)
27,3	4,2	12,6
24,5	4,20	6,42
20,8	2,30	9,11
21,1	2,71	6,41

Infection (%) - Relative to Control

DMSO	WP1130 (Pre-Treatment)	WP1130 (Post-Treatment)
100	15,4	46,2
100	17,1	26,2
100	11,1	43,8
100	12,8	30,4

Infection Rates

DMSO	PR619 (Pre-Treatment)	PR619 (Post-Treatment)
27,3	20,0	22,1
24,5	22,1	23,4
20,8	14,9	19,6
21,1	18,4	20,1

Infection (%) - Relative to Control

DMSO	PR619 (Pre-Treatment)	PR619 (Post-Treatment)
100	73,3	81,0
100	90,2	95,5
100	71,6	94,2
100	87,2	95,3

Huh-7

Infection Rates

DMSO	WP1130 (Pre-Treatment)	WP1130 (Post-Treatment)
88,0	20,1	73,3
87,9	24,1	78,1
74,7	24,3	57,8
73,6	21,4	55

Infection (%) - Relative to Control

DMSO	WP1130 (Pre-Treatment)	WP1130 (Post-Treatment)
100	22,8	83,3
100	27,4	88,9
100	32,5	77,4
100	29,1	74,7

Infection Rates

DMSO	PR619 (Pre-Treatment)	PR619 (Post-Treatment)
88	76,8	83,6
87,9	74,1	82,6
74,7	63,3	64,9
73,6	61,7	67,9

Infection (%) - Relative to Control

DMSO	PR619 (Pre-Treatment)	PR619 (Post-Treatment)
100	87,3	95,0
100	84,3	94,0
100	84,7	86,9
100	83,8	92,3

Vero

Infection Rates

DMSO	WP1130 (Pre-Treatment)	WP1130 (Post-Treatment)
94,3	22,7	90,2
91,1	32,2	80,4
88,3	28,3	61,6
80,7	27,6	77,1

Infection (%) - Relative to Control

DMSO	WP1130 (Pre-Treatment)	WP1130 (Post-Treatment)
100	24,1	95,7
100	35,3	88,3
100	32,0	69,8
100	34,2	95,5

Infection Rates

DMSO	PR619 (Pre-Treatment)	PR619 (Post-Treatment)
94,3	43,4	93,4
91,1	35,5	76,9
88,3	34,2	78,2
80,7	47,5	69,8

Infection (%) - Relative to Control

DMSO	PR619 (Pre-Treatment)	PR619 (Post-Treatment)
100	46,0	99,0
100	39,0	84,4
100	38,7	88,6
100	58,9	86,5

Fig S4 vRNA Fold Change

Hek 293T	
WP1130	
E1	nsp4
0,10338	0,12878
0,09440	0,10688
0,28843	0,33143
0,27316	0,35677
0,14708	0,18091
0,14544	0,17890

Huh-7	
WP1130	
E1	nsp4
0,10282	0,22823
0,17871	0,23401
0,13279	0,29475
0,21154	0,31944
0,34828	0,24858
0,16877	0,24481

Vero	
WP1130	
E1	nsp4
0,04176	0,06778
0,0434	0,06081
0,0528	0,07572
0,0549	0,07175
0,0311	0,04081
0,0365	0,04794

Hek 293T	
PR619	
E	NSP4
0,70097	0,94850
0,75412	0,81865
0,75537	0,98444
0,76871	1,17701
0,71605	1,00315
0,77027	0,80261

Huh-7	
PR619	
E1	nsp4
0,80751	0,82778
0,83974	0,90320
0,95589	1,04336
0,97260	1,05335
0,84287	0,90279
0,9433	1,06622

Vero	
PR619	
E1	nsp4
0,1748	0,22904
0,1734	0,23668
0,1475	0,19322
0,1260	0,16512
0,1307	0,17117
0,1284	0,16815
